# All‐Inside, All‐Soft‐Tissue, Anchor‐Free Meniscal Allograft Transplantation

**DOI:** 10.1002/atn2.70075

**Published:** 2026-05-24

**Authors:** Seksan Kukreja, Kongphop Ratanachai, Wachirawit Penrat, Emmanuelle E. Yap, Bancha Chernchujit

**Affiliations:** ^1^ Orthopedics Department Thammasat University Hospital Klong Luang Pathumthani Thailand; ^2^ Thai Red Cross Society Pathumwan Bangkok Thailand

## Abstract

Meniscal allograft transplantation is an effective surgical option for patients with irreparable meniscal pathology, including chronic root tears, complex or radial tears, and prior meniscectomy. This Technical Note describes an all‐inside, all‐soft‐tissue, anchor‐free arthroscopic technique for meniscal allograft transplantation designed to maintain a truly minimally invasive approach. Standard anteromedial, anterolateral, and mid‐medial collateral ligament portals are used to prepare the meniscocapsular junction and create tibial tunnels for the anterior and posterior roots. The meniscal allograft is sequentially implanted from posterior to anterior, with meniscus‐to‐meniscus hand‐tied sutures providing secure fixation without the use of anchors. The technique allows precise graft adjustment to accommodate minor size mismatch and minimizes risks associated with anchor displacement or failure. Concomitant procedures such as ligament reconstruction or high tibial osteotomy can be performed simultaneously. This anchor‐free, all‐soft‐tissue technique offers a reproducible, cost‐effective alternative to conventional fixation methods while preserving minimal invasiveness and ensuring strong graft integration by continuous fixation of the allograft to the native meniscocapsular junction, anatomic restoration of both meniscal roots, and robust hand‐tied suture fixation that provides controlled tensioning and uniform graft compression without reliance on anchors.

## Graphical abstract

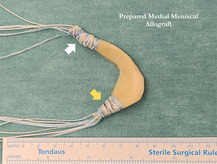

**VIDEO 1:** Arthroscopic medial meniscal allograft transplantation of the left knee. The patient is positioned in the supine semilithotomy position. Graft preparation with anterior and posterior horn suturing is performed concurrently with diagnostic arthroscopy and preparation of the meniscal bed, while preserving the native meniscocapsular junction. Posterior and anterior root tibial tunnels are created under direct visualization. The meniscocapsular junction is presutured using an all‐inside, anchor‐free technique, with the superior suture limbs organized on a punctured sterile ruler to minimize suture tangling. The prepared allograft is introduced through the anteromedial portal and fixed sequentially, beginning with posterior root fixation, followed by reduction of the meniscal body by redirection of the inferior suture limbs, and concluding with anterior root reduction and fixation. Each inferior suture limb is then passed sequentially through the body of the meniscal allograft for definitive circumferential fixation. Final probing and range‐of‐motion assessment confirm stable graft fixation and appropriate graft tracking. Video content can be viewed at https://doi.org/10.1002/atn2.70075.

Meniscal allograft transplantation (MAT) represents the definitive procedure for restoring meniscal function in patients with irreparable meniscal pathology with favorable outcomes and acceptable rates of complication and failure regardless of surgical technique.^1^ Individuals with chronic, non‐repairable posterior root tears, radial tears, complex meniscal injuries, or a history of prior meniscectomy derive substantial benefit from this procedure. Jauregui et al.^2^ reported no significant difference in clinical outcomes between soft‐tissue suture fixation and bone fixation techniques for MAT root fixation. The all‐soft‐tissue approach offers several advantages, including technical simplicity, true minimally invasive access, and secure graft stability.^3^ In this report, we describe an all‐inside, all‐soft‐tissue, anchor‐free MAT technique designed to maintain a minimally invasive profile while eliminating the need for meniscal anchors.

## SURGICAL TECHNIQUE

Video 1 shows the arthroscopic surgical technique for medial meniscus transplantation of the left knee. The same principles can be applied to lateral meniscus transplantation, as well as to concomitant procedures such as high tibial osteotomy or ligament reconstruction.

### Preoperative Evaluation

Indications for MAT include young, active patients with a non‐functional meniscus due to chronic posterior root tears, radial or complex tears, prior meniscectomy, or irreparable meniscal damage. Suitable candidates should have intact or correctable limb alignment, stable or reconstructible ligaments, and no evidence of advanced osteoarthritis. Preoperative evaluation includes standard standing radiographs of the operative knee, full‐length standing (three‐foot) lower limb radiographs, and magnetic resonance imaging (MRI) to assess meniscal deficiency, articular cartilage status, and lower limb alignment.

### Patient Positioning

The patient is placed in a supine, semilithotomy position, with the contralateral leg abducted at the hip and placed into a well‐padded lithotomy‐style legholder, ensuring it remains secure and out of the surgical field. The affected leg is flexed to 90° at the knee, and the posterior thigh is supported, allowing the leg to hang freely off the side of the table. A thigh‐high, well‐padded pneumatic tourniquet is applied on the operative limb. For a medial meniscus transplant, the leg will be abducted and rested on the surgeon's outer hip. The leg is put in figure‐of‐4 fashion for lateral meniscus implant.

### Graft Preparation

Sizing of the allograft is determined using a combination of the Pollard method^4^ and MRI measurements. Meniscal allografts are obtained from the Thai Red Cross Society (Bangkok, Thailand). Each allograft is thawed to room temperature according to the tissue bank's protocol and is provided with proximal tibial bone blocks. The medial meniscus is carefully dissected from the bone.

**FIGURE 1:**
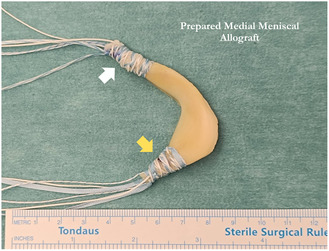
Prepared medial meniscal allograft of the left knee after bone removal. The white arrow indicates the anterior horn, and the yellow arrow indicates the posterior horn of the medial meniscal allograft. A combination of No. 2‐0 Ultrabraid and 3‐mm Ultratape sutures (Smith & Nephew, Andover, MA, USA) is applied to the allograft using modified whipstitch and wraparound techniques at the anterior and posterior horns.

A combination of Ultrabraid No. 2‐0 and 3‐mm Ultratape sutures (Smith & Nephew, Andover, MA, USA) is applied using modified whipstitch and wraparound techniques to the anterior and posterior horns of the allograft, facilitating later fixation with an EndoButton device (Smith & Nephew, Andover, MA, USA) at the tibial site (Figure 1). Prior to implantation, the prepared graft is soaked in 500 mg of vancomycin diluted in 100 mL of normal saline. The posterior aspect of the graft is marked before insertion to ensure proper orientation during arthroscopic placement.

### Arthroscopic Examination and Meniscocapsular Junction Preparation

**FIGURE 2:**
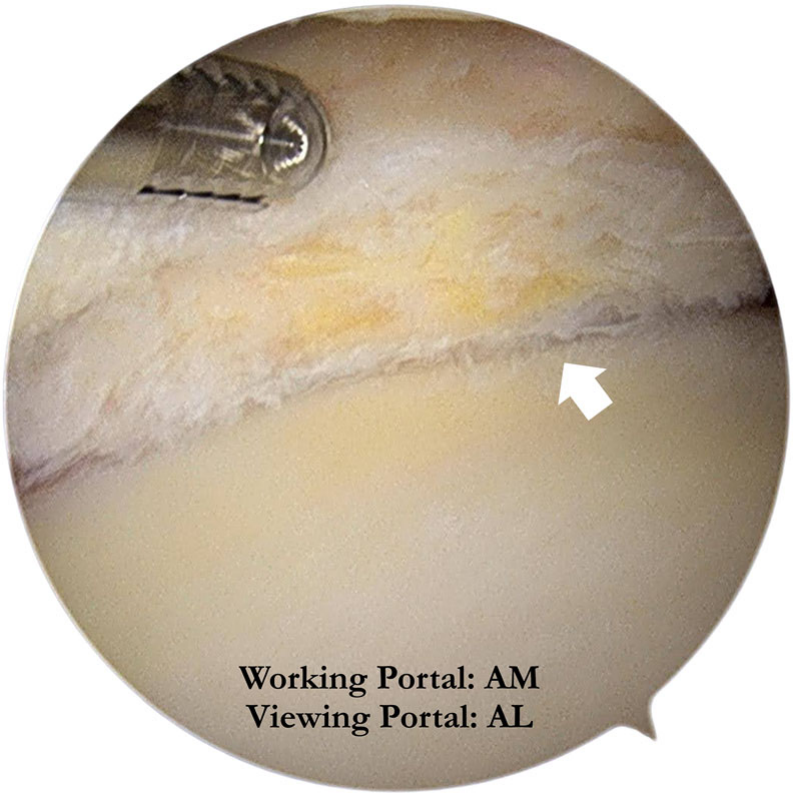
Arthroscopic view of the medial compartment of the left knee, with visualization through the anterolateral portal and instrumentation through the anteromedial portal. The white arrow indicates the remaining meniscocapsular junction after debridement. Approximately 1 to 2 mm of the meniscocapsular junction is preserved to facilitate subsequent repair and integration of the allograft (AL, anterolateral portal; AM, anteromedial portal.)

Standard anterolateral and anteromedial portals are established. A percutaneous partial outside‐in release of the medial collateral ligament (MCL)^5^ is performed to enhance visualization of the medial compartment. A mid‐MCL portal is then created parallel to the MCL, serving as the working portal on the medial side. Debridement of the meniscal bed is carried out, preserving approximately 1‐2 mm of the meniscocapsular junction to facilitate subsequent repair and integration of the allograft (Figure 2). Trephination using an 18‐gauge needle may be employed to promote biological healing at the recipient site.

### Posterior and Anterior Horn Insertion Preparation

**FIGURE 3:**
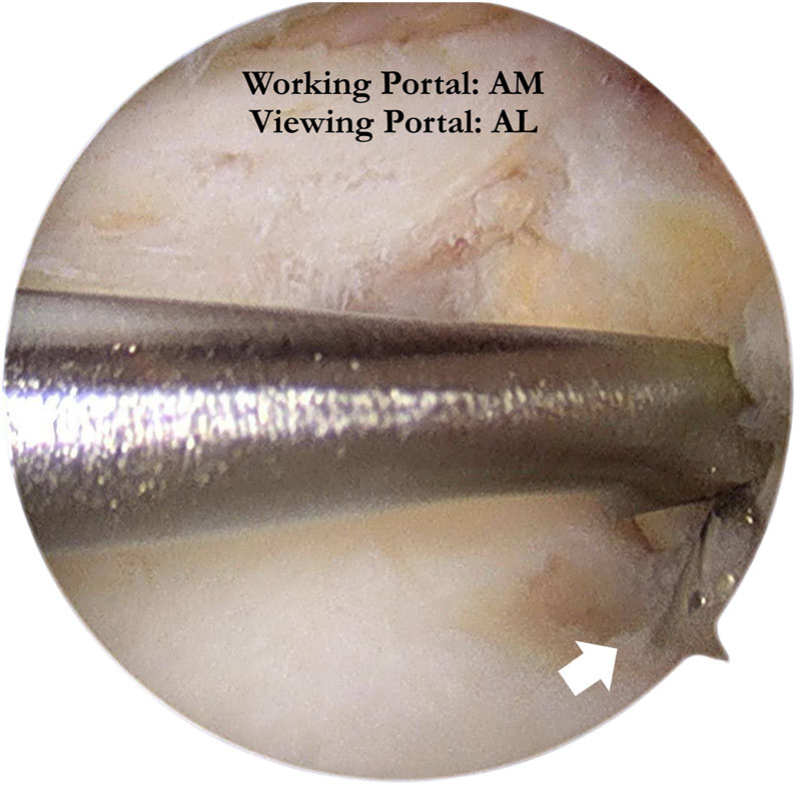
Arthroscopic view of the medial compartment of the left knee, with visualization through the anterolateral portal and instrumentation through the anteromedial portal. The white arrow indicates the tip of the Beath pin used for creation of the posterior root tibial tunnel. A hip guide (Acumed, Hillsboro, OR, USA) is used to create the posterior root tunnel using a 2.4‐mm Beath pin at 50°‐55°, with direct visualization of the emerging pin tip to ensure accuracy. A close‐cup curette is used to protect surrounding soft tissues during drilling. (AL, anterolateral portal; AM, anteromedial portal.)

**FIGURE 4:**
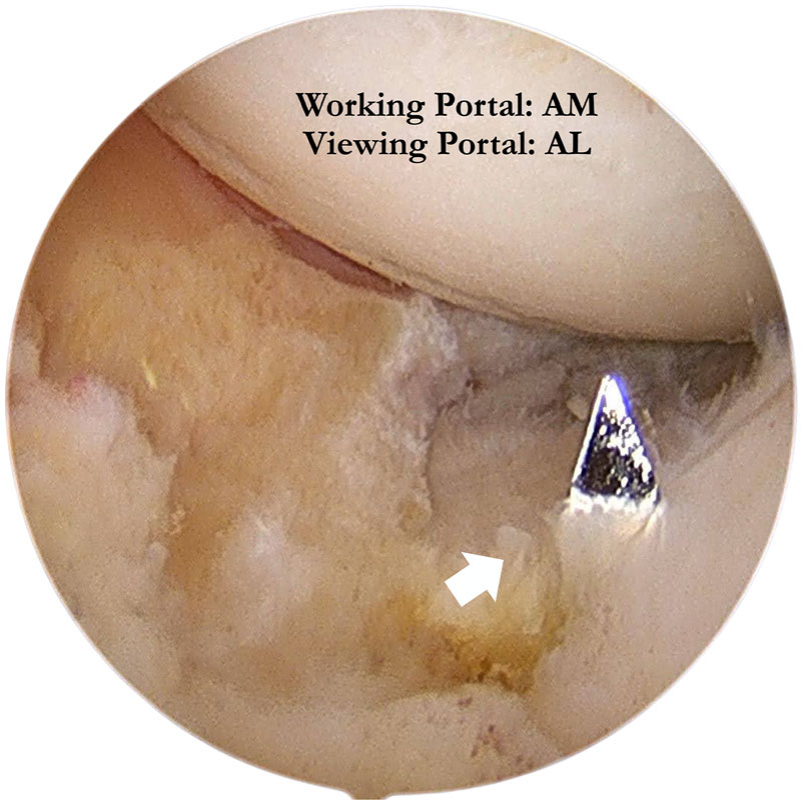
Arthroscopic view of the medial compartment of the left knee, with visualization through the anterolateral portal and instrumentation through the anteromedial portal. The white arrow indicates the tip of the Beath pin used for creation of the anterior root tibial tunnel. The anterior horn tunnel is prepared using a tip‐aiming ACL guide at 60°‐65°. (ACL, anterior cruciate ligament; AL, anterolateral portal; AM, anteromedial portal.)

The insertion sites for the anterior and posterior horns are identified and marked under arthroscopic visualization. A hip guide (Acumed, Hillsboro, OR, USA) is used to create the posterior root tunnel using a 2.4‐mm Beath pin at 50°‐55°, with direct visualization of the emerging pin tip to ensure accuracy. This is followed by drilling with a 4.5‐mm EndoButton reamer (Smith & Nephew, Andover, MA, USA) to create the tibial tunnel. A close‐cup curette is used to protect surrounding soft tissues during drilling. A No. 2‐0 PDS suture (Ethicon, Somerville, NJ, USA) is then passed through the tunnel to serve as a suture shuttle. The anterior horn tunnel is prepared using a tip‐aiming anterior cruciate ligament guide at 60°‐65°, following the same sequence of steps as described for the posterior horn (Figures 3 and 4).

### Meniscocapsular Suturing for Subsequent Allograft Fixation

**FIGURE 5:**
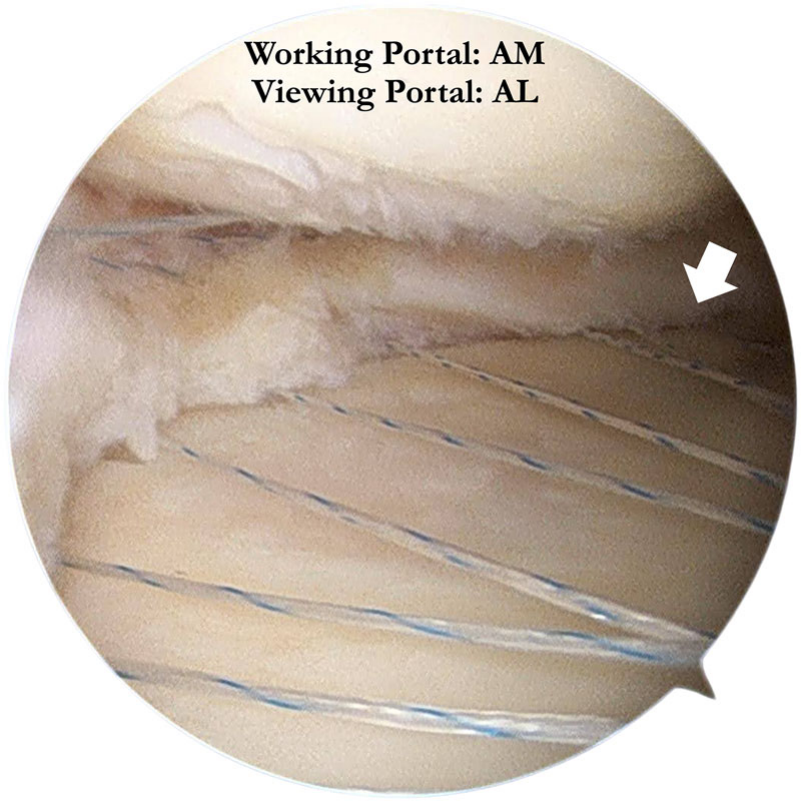
Arthroscopic view of the medial compartment of the left knee, with visualization through the anterolateral portal and instrumentation through the anteromedial portal. The white arrows indicate the inferior suture limbs, which are arranged and passed through the anterolateral portal and reserved for subsequent graft reduction and fixation. (AL, anterolateral portal; AM, anteromedial portal.)

**FIGURE 6:**
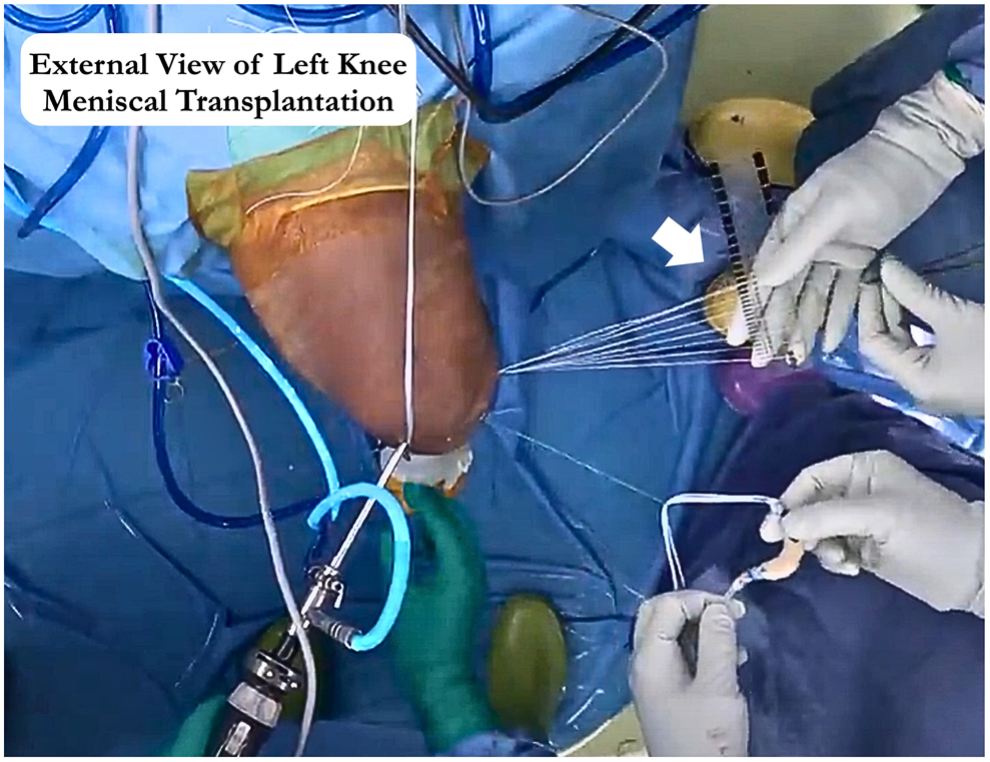
External view of lateral meniscal allograft transplantation in the left knee (example from another case). The superior limbs of the sutures are retrieved through the mid‐lateral portal (mid‐MCL portal for medial meniscal allograft transplantation) and organized sequentially through numbered holes in a punctured sterile ruler (white arrow). (MCL, medial collateral ligament.)

The meniscocapsular junction of the medial compartment is presutured using a combination of the FirstPass Mini and ACCU‐PASS suture devices (Smith & Nephew, Andover, MA, USA). PDS No. 2 sutures (Ethicon, Somerville, NJ, USA) are used initially and subsequently shuttled with Ultrabraid No. 2‐0 sutures (Smith & Nephew). Typically, 8 to 10 sutures are placed to adequately cover the entire compartment. The lower limb of each suture is passed through the anterolateral portal and reserved for subsequent reduction and fixation (Figure 5). The upper limb is retrieved through the mid‐MCL portal and organized sequentially through numbered holes on a punctured sterile ruler, allowing easy identification and controlled fixation of the meniscal allograft during implantation (Figure 6).

### Graft Implantation and Fixation

**FIGURE 7:**
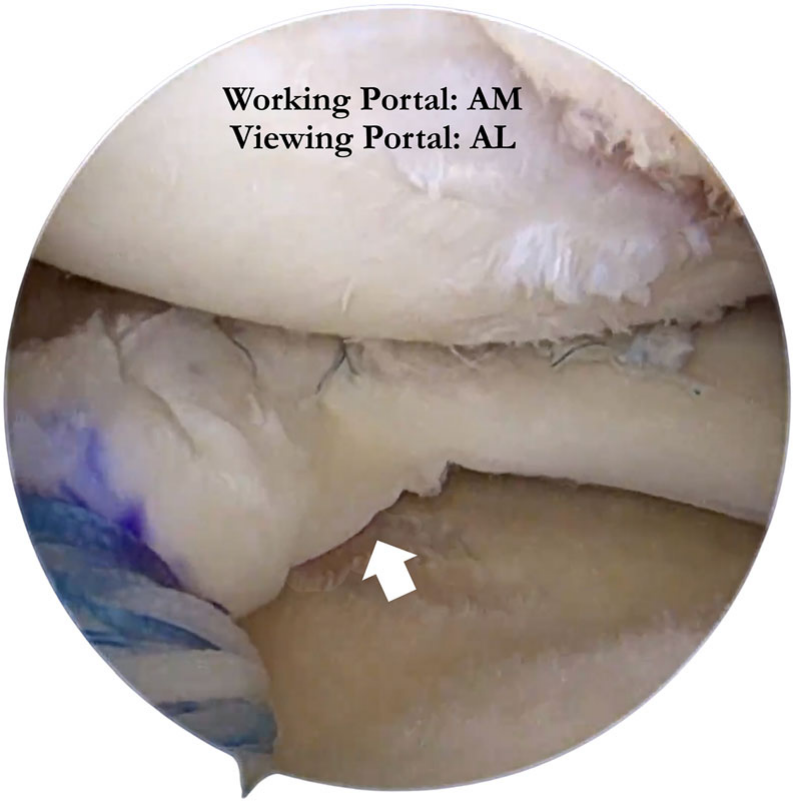
Arthroscopic view of the medial compartment of the left knee, with visualization through the anterolateral portal. The white arrow indicates final implantation and fixation of all‐inside, all‐soft‐tissue, anchor‐free meniscal allograft transplantation. (AL, anteromedial portal; AM, anteromedial portal.)

**FIGURE 8:**
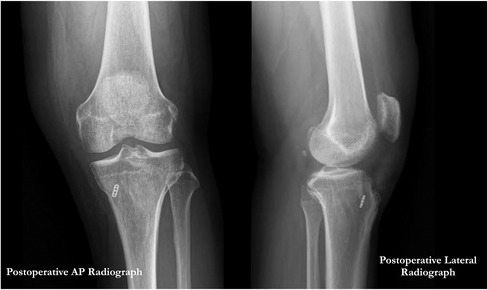
Immediate postoperative anteroposterior and lateral radiographs of the left knee showing satisfactory alignment and restoration of the joint line. (AP, anteroposterior.)

**FIGURE 9:**
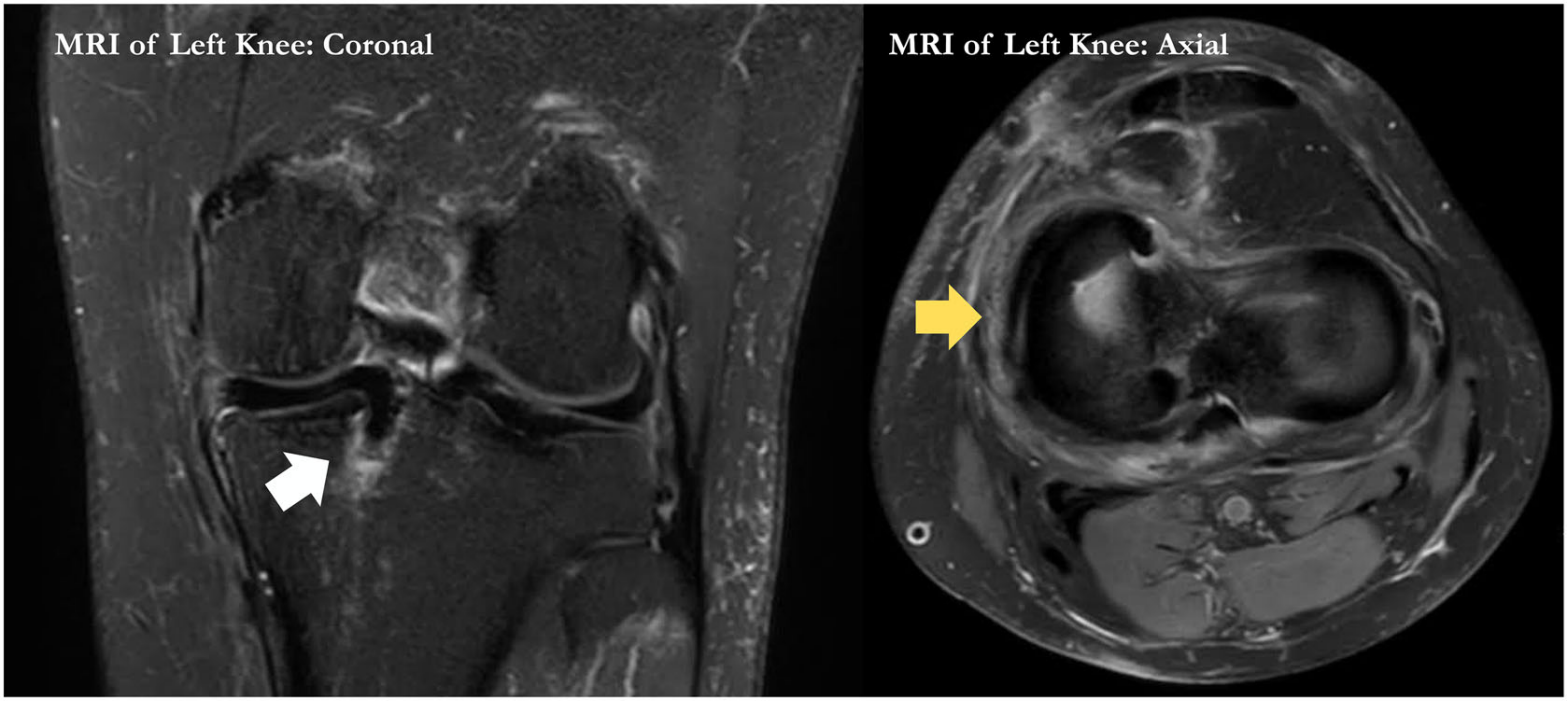
Two‐month postoperative magnetic resonance imaging of the left knee following medial meniscal allograft transplantation, showing appropriate graft alignment. The white arrow indicates secure posterior root fixation, and the yellow arrow denotes a well‐positioned meniscal allograft without evidence of extrusion. (MRI, magnetic resonance imaging.)

Graft implantation follows the principle of sequential fixation, beginning with the posterior root, followed by reduction of the meniscal body, and concluding with anterior root fixation. The graft is introduced into the joint through the anteromedial portal, and the posterior horn sutures are shuttled through the tibial tunnel until the marking on the posterior root reaches the tunnel aperture. Reduction of the meniscal body is achieved by transferring the previously placed lower limbs of the meniscocapsular junction sutures to the mid‐MCL portal, allowing attachment of the allograft to the native meniscocapsular junction. An assistant maintains tension on the redirected suture limbs to facilitate accurate approximation before definitive repair. The anterior root is then implanted by shuttling its sutures through the tibial tunnel using the same technique. Each lower limb of the meniscocapsular sutures is identified sequentially using a knot pusher inserted from the upper limb corresponding to the prearranged numbered ruler system. The lower limb is then passed one by one into the meniscal allograft using an ACCU‐PASS suture device (Smith & Nephew, Andover, MA, USA) with PDS No. 2 sutures (Ethicon, Somerville, NJ, USA) serving as shuttle sutures. Knot tying is performed for each suture using alternating half‐hitches from posterior to anterior in a semicircular fashion. After fixation, the knee is taken through a full range of motion to confirm stability and graft tracking. The anterior and posterior root sutures are tied with Revo knots over an EndoButton (Smith & Nephew, Andover, MA, USA). Final fixation is verified with a probe for stability (Figure 7), and postoperative radiographs and follow‐up MRI at 2 months showed appropriate graft placement and alignment (Figures 8 and 9).

## DISCUSSION

Recent advancements in all‐soft‐tissue medial MAT techniques have been reported over the past decade. Spalding et al.^3^ described an arthroscopic all‐soft‐tissue MAT using a combination of all‐inside and inside‐out sutures, showing reliable clinical outcomes comparable to those achieved with bone plug fixation. Kim et al.^6^ reported a soft‐tissue MAT technique incorporating anterior intermeniscal ligament fixation to minimize meniscal extrusion. Waltz et al.^7^ introduced a segmental medial meniscus allograft transplantation method for focal meniscal defects utilizing all‐inside repair techniques. Richards et al.^8^ presented an all‐soft‐tissue MAT with tape augmentation and centralization to enhance graft stability. Trigg et al.^9^ proposed a hybrid MAT approach employing bone plug fixation for the posterior root and soft‐tissue fixation for the anterior root, designed to achieve secure posterior anchorage while allowing adjustable anterior fixation to accommodate graft mismatch.

Our technique is an all‐inside, all‐soft‐tissue, anchor‐free approach that eliminates the need for meniscal anchor‐based suturing systems, thereby reducing the risk of anchorage failure and minimizing potential irritation from anchor displacement. This all‐soft‐tissue technique offers a truly minimally invasive approach, enabling fine adjustment of graft positioning to accommodate minor discrepancies between graft and recipient site dimensions. The all‐inside, meniscus‐to‐meniscus suturing configuration also helps prevent meniscal extrusion by ensuring an anatomic and secure fixation. Furthermore, concomitant procedures, such as ligament reconstruction, high tibial osteotomy, or osteochondral allograft transplantation, can be performed concurrently using this technique, as reported in the recent literature.^10^, ^11^, ^12^ The absence of anchors not only simplifies the procedure but also contributes to a reduction in overall surgical costs.

Careful attention must be given to suture management and handling throughout the procedure. Although technically demanding, the use of knot pushers and a numbered ruler system can facilitate efficient suture organization and significantly reduce the learning curve. As all sutures in this technique are hand‐tied, potential failure may result from improper placement or inadequate knot‐tying technique. We are currently conducting cohort studies to further evaluate the clinical and radiographic outcomes of this all‐inside, all‐soft‐tissue, anchor‐free MAT technique. The pearls and pitfalls of our technique are summarized in Table 1, and advantages and disadvantages are shown in Table 2.

**TABLE 1:** Pearls and Pitfalls

Pearls
Medial collateral ligament (MCL) release is performed to facilitate access to the medial compartment of the knee
The lower limbs of the sutures are retrieved through the anterolateral (AL) portal before graft introduction and wrapped around the allograft to facilitate reduction prior to definitive repair
All‐inside meniscus‐to‐meniscus suturing helps reduce meniscal extrusion and may prevent future radial tears compared with wraparound sutures

Pitfalls
To avoid suture trafficking, the upper limbs of the sutures are organized through a punctured, numbered sterile ruler
To avoid tunnel jamming, the tibial tunnel for the posterior root is placed more medially, while the anterior root tunnel is positioned laterally
To avoid breakage of the anterior root tibial tunnel, increase the ACL guide angle to create a more vertical and longer tunnel

ACL, anterior cruciate ligament.

**TABLE 2:** Advantages and Disadvantages

Advantages
Eliminates the need for anchors, reducing risks of failure and irritation
Allows fine graft adjustment to accommodate minor size mismatch
An all‐inside meniscus‐to‐meniscus suture provides anatomic and stable fixation, reducing extrusion risk

Disadvantages
Technically demanding in suture handling
Potential failure from inaccurate suture placement or inadequate knot tying

## DISCLOSURES

The authors (S.K., K.R., W.P., E.E.Y., B.C.) declare that they have no known competing financial interests or personal relationships that could have appeared to influence the work reported in this article.
